# Sperm DNA methylation landscape and its links to male fertility in a non-model teleost using EM-seq

**DOI:** 10.1038/s41437-025-00756-y

**Published:** 2025-03-18

**Authors:** Fotis Pappas, Martin Johnsson, Göran Andersson, Paul V. Debes, Christos Palaiokostas

**Affiliations:** 1 Department of Animal Biosciences, Swedish University of Agricultural Sciences, Uppsala, Sweden; 2 Department of Aquaculture and Fish Biology, Hólar University, Sauðárkrókur, Iceland

**Keywords:** DNA methylation, Animal breeding

## Abstract

Differential DNA methylation due to epigenetic phenomena is crucial in regulating gene expression. Understanding the consequences of such differential expression on sperm quality parameters may provide insights into the underlying mechanisms of male reproductive success. Nonetheless, male fertility in fish remains understudied despite its critical importance to overall reproductive success in nature and captivity. This study investigated the DNA methylation landscape in spermatozoa of domesticated Arctic charr (*Salvelinus alpinus*) and its associations with sperm quality parameters. Computer assisted-semen analysis (CASA) was performed in 47 sperm samples of farmed Arctic charr, followed by enzymatic methylation sequencing (EM-seq). Our results showed that the DNA of Arctic charr sperm is highly methylated (mean value of ~86%), though variations were observed in genomic features involved in gene regulation. Methylation at variable CpG sites exhibited regional correlation decaying by physical distance, while methylation similarities among individuals were strongly coupled with genetic variation and mirrored pedigree structure. Comethylation network analyses for promoters, CpG islands and first introns revealed genomic modules significantly correlated with sperm quality traits (*p* < 0.05; Bonferroni adjusted), with distinct patterns suggesting a resource trade-off between sperm concentration and kinematics. Furthermore, annotation and gene-set enrichment analysis highlighted biological mechanisms related to spermatogenesis, cytoskeletal regulation, and mitochondrial function, all vital to sperm physiology. These findings suggest that DNA methylation is a critical and fundamental factor influencing male fertility in Arctic charr, providing insights into the underlying mechanisms of male reproductive success.

## Introduction

Reproductive success is directly linked to fitness in wild populations and is a cornerstone of domestic animal agriculture, ensuring long-term productivity. In aquaculture, the ability to efficiently and reliably yield production stock through a small fraction of breeders is a key feature of the sector’s sustainability (Gjedrem 2012; Nguyen 2016; Gjedrem and Rye 2018; Ren et al. 2020). A reduction in fertility rates following domestication is a general, though not universal, trend in farmed animals, including many captive-reared fish populations. Notably, the causal factors behind this phenomenon remain primarily unknown (Farquharson et al. 2018).

Arctic charr (*Salvelinus alpinus*) is a cold-adapted salmonid fish species (Klemetsen et al. 2003) with high marginal economic value. It is farmed in regions of the far north latitudes, including the Nordic countries and Canada. In Sweden, the oldest selective breeding program for Arctic charr globally is heading towards its 10th selected generation (Palaiokostas et al. 2021). Low and variable reproductive success rates currently hinder expansion (Jeuthe et al. 2019; Palaiokostas et al. 2022; Kurta et al. 2023). A preliminary assessment of genetic parameters using male fertility proxies revealed a moderate heritable component (Kurta et al. 2022), indicating that improvements through selective breeding might be possible.

Epigenetic marks are known to be significant contributors to a plethora of phenotypes in aquatic animals. Such phenotypes include but are not limited to health (Mukiibi et al. 2022), behavior (Berbel-Filho et al. 2020), fertility (Woods Iii et al. 2018; Zhang et al. 2023), sex determination (Tine et al. 2014; Anastasiadi et al. 2018) and domestication (Krick et al. 2021). Most of the relevant research so far has been focusing on the induction of epigenetic alterations due to environmental stressors, such as chemical pollutants and exposure to suboptimal temperatures, with the latter being of additional value under the light of climate change and the possible consequences for both farmed and wild fish stocks. For instance, fish gametogenesis is sensitive to environmental input at the cellular/physiological level (Kumar et al. 2022) and is strictly dependent on epigenetic marks established during gametogenesis (Brionne et al. 2023). Environmentally induced changes to the sperm epigenome, mediated by mechanisms such as DNA methylation, histone modification, and alterations in noncoding RNA profiles, can translate into ejaculate-mediated paternal effects (Evans et al. 2019) that can compromise gametogenesis and/or exert a negative effect on the fitness of subsequent generations (Herráez et al. 2017; Gasparini et al. 2017; Evans et al. 2019; Wellband et al. 2021; Banousse et al. 2024).

Recently, epigenomic profiling of male gametes has been suggested to predict reproductive performance in domesticated animals (Costes et al. 2022). Moreover, deciphering transgenerational inheritance patterns of epigenetic marks and utilizing this information for selective breeding appears promising (Johannes et al. 2009; Varona et al. 2015; David and Ricard 2019). Specifically, theories supporting that the sperm epigenome acts as an additional information carrier across generations and contributes to non-Mendelian inheritance have been gaining popularity (Immler 2018). Thus, potential applications of relevant omics technologies can greatly interest animal breeding (Zhu et al. 2021; Wang et al. 2023).

Despite the growing interest and existing studies on gamete epigenomes of finfish (Woods Iii et al. 2018; Rodriguez Barreto et al. 2019; Wellband et al. 2021; Cheng et al. 2023), knowledge is still sparse; thus, there is considerable room for expansion of the field. High-resolution epigenomic profiling in various aquaculture species and the development of analytical methodology are required to assess the potential for future implementation of such information to assist breeding decisions.

Among the known epigenetic mechanisms, DNA methylation is the most well-studied in fish (Moghadam et al. 2015). Cytosines modified at the 5th carbon position with a methyl group (5mC) and its oxidized form of 5-hydroxymethylcytosine (5hmC) have been shown to have a substantial impact on a wide range of biological processes (Schübeler 2015). The standard route of studying the methylome relies on using sodium bisulfite during sequencing library preparation, resulting in the conversion of unmethylated cytosines to uracils that are read during sequencing as thymines. A recent technology for methylome-wide sequencing libraries, named enzymatic methyl-seq (EM-seq), relies purely on the enzymatic treatment of the DNA for mapping 5mC and 5hmC (Vaisvila et al. 2021). Opposed to the current golden standard whole-genome bisulfite sequencing (WGBS) approach, EM-seq avoids the chemically detrimental DNA template bisulfite reaction. As a result, enzymatic approaches require a lower sequencing coverage than WGBS while being less prone to GC content bias (Han et al. 2021; Guanzon et al. 2024).

Sperm DNA methylation marks of Arctic charr from the Swedish breeding program were the focal point of this study. These marks were studied at high resolution, and associations were investigated with indirect measures of male fertility, such as sperm concentration and motility parameters. By deciphering the epigenetic landscape of Arctic charr spermatozoa, we aimed to elucidate underlying mechanisms that may contribute to the observed variability in reproductive success rates. Understanding such factors is crucial, as it can lead to more informed breeding strategies that could ultimately enhance reproductive performance and sustainability in Arctic charr farming. Lastly, insights gained from this study may offer new avenues for mitigating fertility issues in salmonids and potentially in other species.

## Materials and methods

### Sampled animals and phenotypic recordings

The studied animals originated from the 8th generation of the Swedish Arctic charr breeding program and were of known pedigree. All fish were reared in one indoor concrete tank with a flow-through water supply and ambient water temperature. Artificial stripping was performed when the fish were 3^+^ years old. Before sample collection, fish were anesthetised using *MS-222* (Sigma-Aldrich, Darmstadt, Germany). Milt samples were collected through manual stripping and stored at 4 °C. Phenotypes related to sperm quality were measured on the same day. Thereafter sperm samples were treated for longer term storage (post 6 h) at −20 °C using absolute ethanol as fixative.

Sperm motility and related kinematic parameters were recorded as previously described by Kurta et al. (2022) using a computer-assisted sperm analysis (CASA) system equipped with the SCA^®^ Motility imaging software (Microptic S.L., Barcelona, Spain). CASA measurements for each sample were taken 2–3 times using 20 µm-depth slides with two counting chambers (CellVision, Heerhugowaard, The Netherlands).

The following CASA parameters were recorded at 15 s immediately after activation with water: proportion of total motility, total progressive motility, total medium motility and total rapid motility together with kinematic metrics including average path velocity (VAP, μm/s), curvilinear velocity (VCL, μm/s) and straight-line velocity (VSL, μm/s). The image capture configurations used for analysis were set to a frame rate of 100 fps (50 frames), while the minimum velocity for motile sperm was set to VCL ≥ 20 µm/s. Additionally, sperm concentration (SC, ×10^6^cells/mL) was measured using the NucleoCounter® SP-100™ (ChemoMetec A/S, Allerod, Denmark). Finally, the sampled animals’ body weight (g, ±10 g) and total length (mm, ±10 mm) were recorded. An exploratory analysis of the recorded data was performed using the R statistical programming language (R Core Team 2023).

### DNA extraction and sequencing library preparation

Genomic DNA was extracted from the collected milt using a salt-based precipitation method (Manousaki et al. 2016). Briefly, 5 μl of milt following centrifugation (13,000 *×* *g*; 1 min) and removal of supernatant was digested overnight at 55 °C using a lysis solution containing 400 μL SSTNE (50 mM Tris base, 300 mM NaCl, 0.2 mM each of EGTA and EDTA, 0.15 mM of spermine tetrahydrochloride, and 0.28 mM of spermidine trihydrochloride; pH 9; Sigma-Aldrich, Darmstadt, Germany), 10% SDS (Bio-Rad, Hercules, CA, USA), and 10 μL proteinase K (Thermo Fisher, Vilnius, Lithuania) (20 mg/mL). After adding 5 μL RNase A (Thermo Fisher, Vilnius, Lithuania) (2 mg/mL), each sample was incubated at 37 °C for 60 min. Protein precipitation was performed by adding 0.7 volume of 5 M NaCl (Sigma-Aldrich, Darmstadt, Germany) with 400 μL of the supernatant transferred to a new microtube. DNA was precipitated using an equal volume of isopropanol and following centrifugation (Pico 21, Thermo Fisher Scientific, Waltham, MA, USA) at 14,000 *×* *g* for 5 min. The DNA pellet was cleaned through overnight incubation with 75% ethanol and dissolved in 30 μL of 5 mM Tris (pH 8.0; Sigma-Aldrich, Darmstadt, Germany). The DNA concentration and quality metrics were recorded using a NanoDrop 8000 (Thermo Fisher Scientific) spectrophotometer, agarose gel electrophoresis, and a Qubit 2.0 Fluorometer (Invitrogen, Carlsbad, CA, USA).

Library preparation was performed using the NEBNext Enzymatic Methyl-seq kit (New England Biolabs, Ipswich, MA, USA). Compared to bisulfite-based approaches, the EM-seq libraries use TET2 and an oxidation enhancer to protect methylated cytosines from downstream deamination with APOBEC. Overall, 48 EM-seq libraries were constructed, including two controls, one fully CpG methylated DNA from pUC19 and one unmethylated lambda phage DNA. Paired-end 150 cycles sequencing was performed in 6 lanes of Novaseq 6000 S4 flow cells. In each sequencing run, a 10% spike-in phage PhiX was added. Library preparation and sequencing were performed at the Swedish National Genomics Infrastructure Center (Science for Life Laboratory, Uppsala University, Sweden). The sequenced reads were deposited in the National Centre for Biotechnology Information repository as fastq files under project ID PRJNA803600.

### Bioinformatic analysis for inferring methylation levels

Raw reads were filtered and trimmed using fastp v0.23.2 (Chen et al. 2018) and were analyzed with Bismark v0.23.1 (Krueger and Andrews 2011) for methylation inference. More specifically, reads passing QC were aligned to a modified *Salvelinus sp*. reference genome (NCBI ASM291031v2). The latter was performed using the *bismark_genome_preparation script* to account for the conversion of unmethylated cytosines to thymines due to the enzymatic reactions during library preparation. Thereafter, duplicated reads were removed using the *deduplicate_bismark* script, followed by inferring methylation levels in CpG, CHG and CHH (where H represents A, C of T) contexts using the *bismark_methylation_extractor* script. All the above data processing steps were conducted using Snakemake v7.9 (Mölder et al. 2021) and can be accessed through https://github.com/pappasfotios/AC_EMseq/tree/main/Reads_to_meth_pipeline. The alignment step of the same computational workflow was performed separately for the nucleotide sequences corresponding to the methylated (pUC19 cloning vector, NCBI M77789.2) and unmethylated (Lambda phage, NCBI GCF_000840245.1) controls.

To avoid biases in the inferred methylation levels due to transitions, the aligned sequence data were used to call single nucleotide polymorphisms (SNPs) using a double-masking approach (Nunn et al. 2022) implemented through Revelio (https://github.com/bio15anu/revelio) and FreeBayes v1.3.2 (Garrison and Marth 2012). A multi-sample marker set in variant call format (VCF) was produced. CpG sites corresponding to SNPs (transitions) were detected with bcftools v1.9 (Li 2011; Danecek et al. 2021) and excluded from downstream methylation analysis. Methylation proportion values for CpGs in different genomic features were extracted using the assembly annotation files in GFF format and utilities provided by the packages bsseq v1.36.0 (Hansen et al. 2012), comethyl v1.3.0 (Mordaunt et al. 2022) and GenomicRanges v1.52.1, (Lawrence et al. 2013) developed in the R statistical programming language (R Core Team 2023). Sets of genomic regions corresponding to gene bodies, promoters defined as regions up to 1Kb upstream of transcription start sites (TSS), exons, introns, first introns and intergenic regions were detected with regard to the genomic annotation file accompanying the assembly and were included in the analysis together with CpG islands and shores that were identified by EMBOSS/newcpgreport (Larsen et al. 1992) and BEDTools v2.31.1 (Quinlan and Hall 2010). In addition, a set of high-quality CpGs with variable methylation in the population was determined as follows: (1) standard deviation of methylation proportion values (methSD) > 0.1, (2) locus coverage ≥10, (3) call-rate = 100%.

### Decay of methylation with physical distance

The level of comethylation at CpGs with variable methylation levels (methSD > 0.1) and its decay with physical distance was analyzed by randomly sampling genomic bins of different sizes. For short-range comethylation, bins ranging from 100 to 1000 bases were used, while for medium-range comethylation, bins from 10 Kb to 100 Kb were sampled. These analyses were conducted using helper functions in R/regioneR v1.32.0 (Gel et al. 2016) and involved the calculation of average absolute correlations (Pearson’s correlation coefficients) within the sampled bins.

### CpG methylation variation vs. genetic variation

To assess the dependence between genetic and epigenetic relationships, we constructed a methylation relationship matrix (MRM), an additive genetic relationship matrix (A_MAT_) and a genomic relationship matrix (GRM). Considering the CpG methylation values filtered as described above, a standardized matrix named M was constructed, with rows corresponding to individuals and columns corresponding to high-quality variable CpGs. Thereafter, a methylation relationship matrix (MRM) was constructed as follows (Zhang et al. 2019):

$${MRM}=\frac{1}{n}M{M}^{T}$$

where ${M}^{T}$ is the transpose of the methylation matrix, and *n* is the number of loci.

The A_MAT_ was constructed using R/pedigree v1.4.2 (Coster 2022), while the GRM was computed with PLINK v1.9 (Chang et al. 2015) and the *–make-rel* function. In the case of GRM, the VCF file containing information for genetic variants was filtered for biallelic markers with a call rate of 100% and a minor allele frequency of at least 20% using VCFtools v0.1.16 (Danecek et al. 2011). Pearson’s correlation coefficients between the off-diagonal elements of MRM and A_MAT_ or GRM were used to assess dependencies.

### Annotation-dependent association between methylation levels and sperm quality traits

The R package comethyl v1.3.0 (Mordaunt et al. 2022) was employed to initially identify comethylated regions (regions with correlated CpG methylation levels) and construct weighted comethylation networks. Analyses were performed separately on either promoters, CpG islands or first introns, methylation of which has been previously associated with repression of transcription (Anastasiadi, Esteve-Codina et al. 2018). Post filtering (methSD >0.05, coverage ≥10) averaged methylation in the selected regions was used to construct comethylation networks and investigate potential associations with the recorded sperm quality traits. In brief, the algorithm adjusted methylation data for the top Principal Components, clustered regions by Pearson’s correlation, selected soft-power thresholds for network construction and detected comethylation modules using hierarchical clustering based on network topologies (Langfelder and Horvath 2008; Mordaunt et al. 2022). Eigennode values corresponding to modules were extracted for each sample, and module-trait associations were assessed using Pearson’s correlations. Statistical significance was determined using Bonferroni adjustments for multiple testing (*p* < 0.05).

Genomic regions corresponding to modules that passed the assigned significance thresholds were further investigated regarding their gene content using a custom script (https://github.com/pappasfotios/AC_EMseq/blob/main/Utilities/annotate.sh) that utilizes BEDtools (Quinlan and Hall 2010) functions and DIAMOND BLASTx (Altschul et al. 1990; Buchfink et al. 2015) searches against zebrafish (GRCz11) and human (GRCh38) protein libraries. For promoters and first introns, the neighboring gene bodies were listed. At the same time, in the case of CpG islands, the possibility of long-range interactions was considered by including genes within 10 Kb upstream and downstream of hits. Gene-list groups were analyzed collectively according to trait category and correlation sign. Gene-set enrichment analysis and pathway analysis were performed on the web interface of the Enrichr suite (Chen et al. 2013; Kuleshov et al. 2016; Xie et al. 2021). In particular, Enrichr was used for the enrichment of human and FishEnrichr for zebrafish homologs, respectively. Enriched BioCarta (Rouillard et al. 2016) and Panther (Mi et al. 2013; Thomas et al. 2022) pathways were extracted from the former while co-expression gene ontology terms for cellular components and biological processes were indicated from the latter.

### Annotation-free association scan between methylation levels and sperm quality traits

For annotation-free scans, clusters of adjacent CpGs were considered using conservative criteria. Groups of at least five CpGs located within 150 bp at most from each other, with a methylation-value standard deviation of more than 0.1 and a coverage of at least 10× could form a cluster. Two case-control studies were performed. More specifically, separate subsets of fish with the 20 lowest and 20 highest observed values for VCL (actual path velocity was assumed to be directly linked with flagellar motor activity) and sperm concentration were considered as respective cases and controls. Welch’s *t*-tests were then performed to test for associations with methylation levels at each individual CpG cluster. *p* value adjustment using the Benjamini–Hochberg method (Benjamini and Hochberg 1995) and a Bonferroni-corrected significance level of 5% were employed to reduce expected Type I errors. Hit loci and their 50Kb flanking regions were subsequently searched for residing genes.

## Results

### Exploratory analysis of recorded sperm quality traits

Considerable variation in milt quality was detected, as revealed by sperm concentration and kinematic parameters (Table 1). An outlying value of 13,536 ×10^6^ cells/mL was detected for sperm concentration, exceeding the expected distribution range. To avoid distortion in downstream analysis, this value was replaced with 10,000 ×10^6^ cells/mL. The replacement value was chosen as a more representative upper bound, accounting for approximately three standard deviations from the respective mean (after replacement).

**Table 1:** Descriptive statistics of phenotypes recorded on the studied male charrs (*n* = 47).

	Min	Median	Mean	Max	Standard deviation
Length (cm)	48.00	54.50	54.78	61.50	2.93
Weight (Kg)	1.51	2.80	2.94	4.40	0.61
Concentration (×10^6^ cells/mL)	1572	3628	4117	10,000	1961.6
Total motility (proportion)	0.34	0.89	0.84	0.99	0.13
Total progressive (proportion)	0.10	0.44	0.46	0.93	0.22
Total rapid (proportion)	0.01	0.09	0.13	0.39	0.11
Total medium (proportion)	0.08	0.33	0.33	0.67	0.14
VCL (μm/s)	53.08	81.74	85.36	134.93	19.66
VAP (μm/s)	33.19	58.24	60.78	101.34	18.52
VSL (μm/s)	19.63	42.70	44.62	71.13	15.06

Length and weight were measured to the nearest millimeter and gram, respectively.

**Fig. 1:**
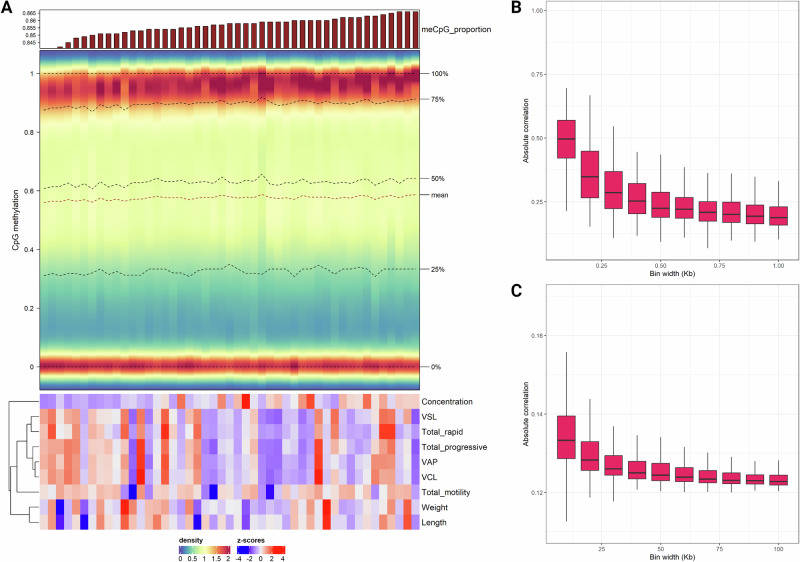
Visualizations for exploratory analysis of sperm DNA methylation landscape. **A** Complex density heatmap for individual distributions of methylation proportion values in the 112,418 CpG sites with variable methylation. The top annotation is a barplot representing global CpG methylation levels for the corresponding charrs, while the bottom annotation is a heatmap with *z*-scores for various sperm quality parameters. **B** Short-range comethylation, quantified as mean absolute correlations in sampled genomic bins ranging from 0.1 Kb to 1 Kb. **C** Medium-range comethylation, quantified as mean absolute correlations in sampled genomic bins ranging from 10 Kb to 100 Kb. The figure was created with R/Complexheatmap v2.16.0 (Gu 2022), R/ggplot2 v3.4.4 (Wickham 2016) and BioRender.com.

As expected, traits corresponding to sperm motility and velocity parameters commonly displayed a strong positive correlation with each other (low annotation heatmap, Fig. 1A). While the same was true for growth-related traits, sperm concentration mostly showed low negative correlations with the different phenotypes.

### DNA methylation landscape of Arctic charr sperm

Quality control by examining DNA methylation levels in samples, as well as positive and negative controls, revealed one sample characterized by artificial, non-biological hypermethylation (Table S1). This outlier was probably a result of failed cytosine conversion and was thus excluded from downstream analyses. The global proportion of methylation in the CpG context ranged from 84.1% to 86.6%, with a median value of 85.8% (Fig. 1A) in the studied individuals. For CHH and CHG contexts, proportions ranged from 0.3% to 0.4%, with a median of 0.3% (Table S1). In total, ~17 million CpG sites were examined with relevant distributions per annotation feature shown in Fig. S1. CpG islands and shores exhibited high and consistent methylation compared to the other examined sets. On the other hand, promoters, first intron and exons notably showed more variability in methylation levels (top panel of Fig. S1). A subset of 112,418 CpGs with variable methylation (methSD >0.1, coverage ≥10, 100% call rate) were retained for downstream analysis. The distribution of the respective methylation proportion values in this set was trimodal for each charr with modes for hypomethylated, hypermethylated and semimethylated states (respectively defined as the lower, middle and upper third intervals of the 0 to 1 range, Fig. 1A). The latter corresponded to a low mode for methylation proportions around 0.5. Epiallelic state at these CpGs showed regional comethylation measured as mean absolute correlation in bins of varying length (specifically in ranges of 0.1 to 1 Kb and 10 to 100 Kb). This phenomenon seems to follow an exponential decay pattern by physical distance at both short and medium ranges (Fig. 1B, C).

### Epigenetic-genetic relationships

**Fig. 2:**
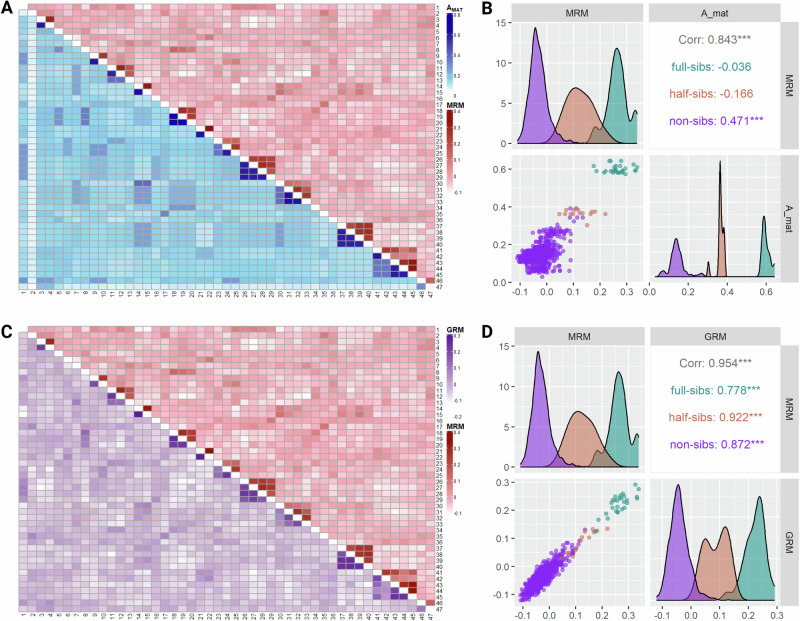
Epigenomic–genetic relationships. **A** Heatmap visualizing off-diagonal elements of A_mat_ (lower triangular heatmap) and MRM (upper triangular heatmap). **B** Facet displaying correlation coefficients, scatterplot and kernel-smoothed densities for MRM and A_mat_ off-diagonal elements by kinship. **C** Heatmap visualizing off-diagonal elements of GRM (lower triangular heatmap) and MRM (upper triangular heatmap). **D** Facet displaying correlation coefficients, scatterplot and kernel-smoothed densities for MRM and GRM off-diagonal elements by kinship. Figure was created with R/Complexheatmap v2.16.0 (Gu 2022), R/GGally v2.2.0 (Schloerke et al. 2024) and BioRender.com.

Methylation similarities computed in the methylation relationship matrix (MRM) recovered the pedigree structure represented by the additive genetic relationship matrix as visualized by the lower and upper triangular heat maps in Fig. 2A. The same pattern was observed also when the MRM was compared against the genomic relationship matrix (GRM) (Fig. 2C). In particular, the off-diagonal elements of the MRM exhibited a strong positive correlation of 0.84 and 0.95 with the respective elements of the A_MAT_ and the GRM (Fig. 2B, D, respectively).

### Annotation-dependent association using comethylation networks

**Fig. 3:**
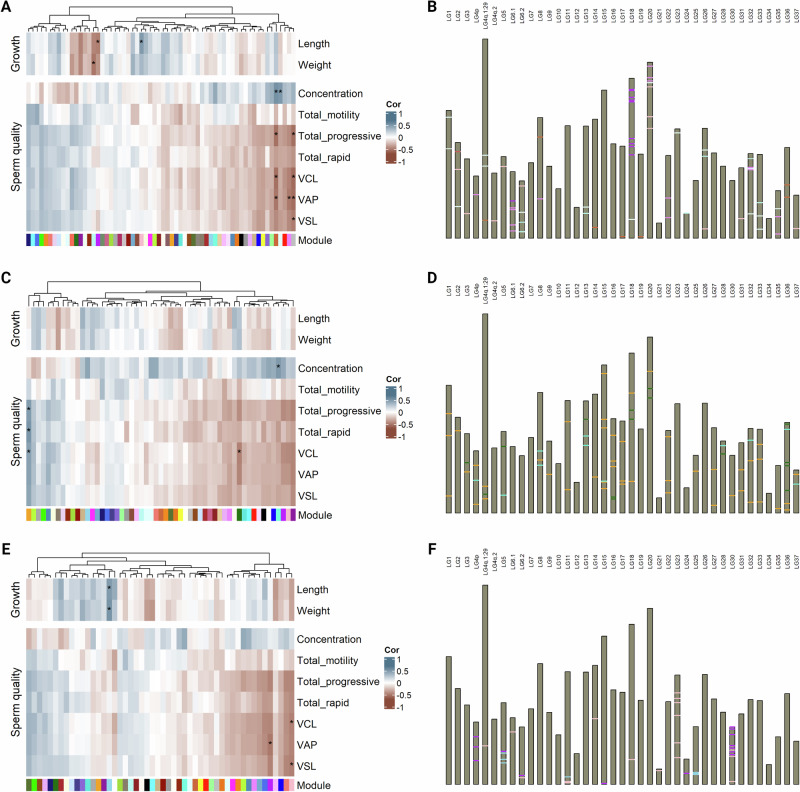
Signals from comethylation-network analyses. **A** Heatmap with correlations between phenotypic vectors and module eigennodes yielded from promoter regions analysis. **B** Pseudo-karyogram indicating promoter regions corresponding to significantly associated modules. **C** Heatmap with correlations between phenotypic vectors and module eigennodes yielded from CpG island analysis. **D** Pseudo-karyogram indicating CpG island regions corresponding to significantly associated modules. **E** Heatmap with correlations between phenotypic vectors and module eigennodes yielded from first intron analysis. **F** Pseudo-karyogram indicating first intron regions corresponding to significantly associated modules. Heatmap cells marked with * highlight significant associations (Bonferroni-adjusted 5%), while band colors in pseudo-karyograms correspond to respective module colors. Figure was created with R/Complexheatmap v2.16.0 (Gu 2022), R/ggbio v1.48.0 (Yin et al. 2012) and BioRender.com.

Comethylation networks were constructed for three different genomic features, namely promoters defined as 1 Kb upstream regions relative to transcription start sites, CpG islands and first introns that, after filtering accounted for 4398, 1178 and 1031 genomic regions, respectively. Comethylation network analysis yielded 62 modules for promoter regions. Four of those modules were significantly correlated with sperm quality traits (Fig. 3A). One of those modules depicted as “gray” accounted for regions not assigned to any comethylation network. These modules, had an opposite correlation sign for sperm concentration and sperm motility/velocity traits. Specifically, methylation in two of these modules was positively correlated with sperm concentration, while negative correlations were observed in the case of sperm motility and velocity. For CpG islands, the analysis returned 55 comethylation modules. Similar to promoters, one positively correlated module appeared for sperm concentration, and another one was negatively correlated with curvilinear velocity (VCL). Furthermore, a single module was significantly and positively correlated with two sperm motility and one sperm velocity parameters. Again, all statistically significant modules had opposing correlation signs for sperm concentration and sperm motility/velocity (Fig. 3C). Lastly, when considering first introns, 50 modules were detected. In this case, VCL, VAP and VSL displayed strong negative correlations with two modules (Fig. 3E).

**Fig. 4:**
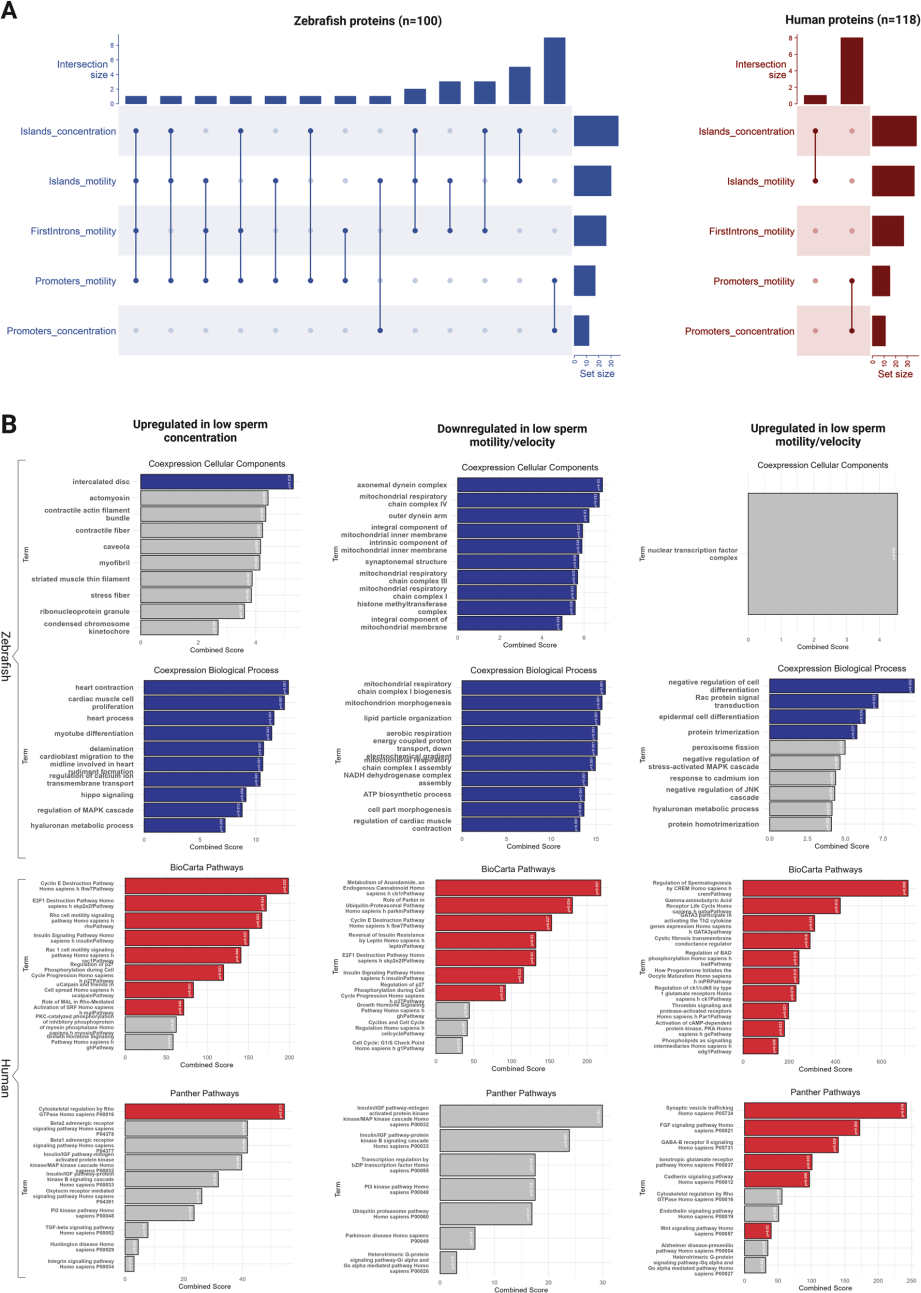
Visualization of signal annotation and gene-set enrichment analysis. **A** UpSet plots for zebrafish and human homologs indicating overlaps between gene-sets yielded from associations of CpG island comethylation modules with concentration and motility-related traits, first intron modules with motility-related traits and promoter modules with both concentration and motility-related parameters. **B** Panels showing the top ten enrichment terms across multiple categories, including Coexpression-Predicted Cellular Components and Coexpression-Predicted Biological Processes yielded by zebrafish homolog-sets (submitted to FishEnrichr) and pathways from BioCarta and Panther yielded by human homolog-sets (submitted to Enrichr). The left column focuses on the upregulated sets in low sperm concentration, the middle column represents downregulated sets in low motility/velocity and the right column highlights results for upregulated components in low motility/velocity. Ranking of terms and bar heights represent the respective Enrichr and FishEnrichr combined scores, while gray bars indicate terms that failed to pass the significance level of 5%. Figure was generated with R/Complexheatmap v2.16.0 (Gu 2022), R/ggplot2 v3.4.4 (Wickham 2016) and BioRender.com.

A considerable amount of overlap in the resulting annotated gene lists was observed between the different significant modules (Fig. 4A), mainly due to opposing correlation signs for sperm concentration and sperm motility/velocity traits. Despite the expected noise being present in the results, annotation revealed multiple candidate genes with strong biological relevance to male fertility. Among the noteworthy findings are genes encoding serine/threonine kinases, including a testis-specific homolog and *DMRT2* (Doublesex mab-3 related transcription factor 2). The latter is a key regulator of sexual development (Feng et al. 2021), while influencing also sex reversal (Raymond et al. 1999), testicular formation and the overall gonadal function (Shi et al. 2014; Wang et al. 2023) across diverse species. Additionally, genes coding for proteins such as Inhibin A, which modulates the production of follicle-stimulating hormone (FSH) and receptors for oxytocin and relaxin, hormones involved in the reproduction cascade, were identified. The presence of follicle growth factor and components of the WNT signaling pathway, such as WNT3 and the transcription factor FOXA1, further point to a network of reproduction-related regulatory mechanisms. Moreover, additional candidate genes coding for proteins such as the insulin receptor, a selenoprotein, several ion pumps, solute carriers and an adenylate cyclase were also present in the set with candidate-genes associated with sperm physiology and thus likely to play a role in fertility.

Under the assumption that CpG methylation in all three genomic features (promoters, CpG islands, first introns) is associated with the silencing of RNA polymerase II-mediated transcription of protein coding genes the signal list was divided into three categories: (1) upregulated genes in low sperm concentration, (2) downregulated genes in low sperm motility/velocity and (3) upregulated genes in low sperm motility/velocity (only observed for CpG islands, Fig. 3C). These hit lists were further investigated with regard to functional genomic information by gene-set enrichment analysis that yielded multiple terms for each category (Fig. 4B). The “upregulated in low sperm concentration” gene set mostly recovered ontology terms affiliated with heart functions and a number of pathways related to cytoskeleton remodeling, cell cycle and Rac1 cell motility. Additionally, the ubiquitin-proteasome system, insulin signaling, and inflammation mediated by chemokine and cytokine signaling were also highlighted. For the “downregulated in low sperm motility/velocity” set, highly relevant terms were enriched for cellular components and biological processes. More specifically, terms affiliated with mitochondria, energy release through respiratory chains and axonemal assembly uncovered direct links to spermatozoa morphogenesis and physiology. Moreover, anandamide metabolism was the pathway that ranked first among other significant ones for the “upregulated in low sperm concentration” gene set. Lastly, the “upregulated in low sperm motility/velocity” gene set were enriched with biological process terms affiliated to regulation of cell differentiation, Rac signal transduction and protein trimerization. Pathways including “Regulation of Spermatogenesis by CREM” alongside key neural signaling pathways such as GABA receptor life cycle and synaptic vesicle trafficking were marked. Other significant pathways enriched by this gene-set involved cell survival mechanisms such as the regulation of BAD phosphorylation, cadherin signaling and cytoskeletal regulation by Rho GTPase.

### Annotation-free association scan

**Fig. 5:**
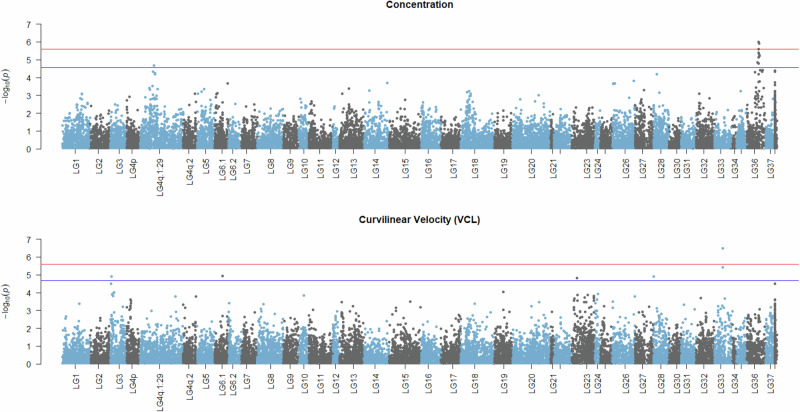
Manhattan plots representing methylome association scans for sperm concentration and curvilinear velocity (VCL). Clusters of adjacent CpGs are grouped in their corresponding linkage groups along the horizontal axis with respect to physical position. The negative decimal logarithms of the relevant *p* values are used to create a gradient along the vertical axis. Red horizontal lines indicate methylome-wide significance threshold corresponding to a Bonferroni-corrected 5% while blue horizontal lines highlight Benjamini–Hochberg 5% cut-offs. Figure was created using R/qqman v0.1.9 (Turner 2018).

The annotation-free case-control study identified signals for both sperm concentration and VCL. Those signals mainly were concerning increased methylation in the case of low sperm quality. In particular, seven signals (three below the Bonferroni-adjusted threshold) were associated with higher methylation in the low sperm concentration group, while another three were less methylated in the same individuals (Fig. S2A). For VCL, five signals (one below the Bonferroni-adjusted threshold) were identified as more methylated in low velocity, while one signal showed decreased methylation in the same group (Fig. S2B). As visualized in the Manhattan plots (Fig. 5), some of the aforementioned signals are co-localized, revealing chromosomal segments of associated CpGs. This pattern was mostly profound in the case of sperm concentration where two peaks, one in LG36 and one in LG4q.1:29, were apparent.

Regarding the genomic information contained within the highlighted loci and their 50Kb flanking regions, 32 protein-coding candidate genes were identified and listed in Table 2. The top hit for sperm concentration was at ~28 Mb of LG36, neighboring a gene body coding for cytochrome c1-like. However, in terms of proximity, the signal is located around the transcription start site of a long non-coding RNA. A search with the Basic Local Alignment Tool (Camacho et al. 2009) revealed sequence similarity to *DHRSX* (dehydrogenase/reductase X-linked), a gene that resides in the pseudoautosomal region (PAR) of mammalian X chromosomes. Interestingly, more homologs of known PAR and PAR-neighboring genes like *AKAP17A* and *ARSH* were found in the same segment of LG36. The secondary peak for sperm concentration on LG4q.1:29 contained three candidate genes encoding proteins with functions related to neurotransmission, sterol metabolism and cell-cycle regulation. For VCL, the highest signal primarily involved genes with functions related to DNA replication, ubiquitination and actin skeleton dynamics.

**Table 2:** Summary of genomic locations and annotated protein-coding genes residing within and 50 Kb upstream and downstream of association signals.

Linkage group	Start position (bp)	End position (bp)	Protein-coding genes within signal and 50 Kb on either side
Sperm concentration			
LG4q.1:29	28895509	28896525	•Substance-P receptor-like, •Scavenger receptor class B member 1, •Cell cycle and apoptosis regulator protein 2
LG36	23528620	23529340	•Sodium/myo-inositol cotransporter, •Potassium voltage-gated channel subfamily E member 2, •Calsequestrin-2-like, •ATP synthase subunit O (mitochondrial), •Sodium/myo-inositol cotransporter-like, •Protein dopey-2, •Vang-like protein 1
LG36	26070033	26070330	•Complement C1q-like protein 2, •Homeobox protein engrailed-1-B-like, •Insulin-induced gene 2 protein
	26070503	26070630	
	26070820	26071374	
	26072622	26073290	
	26082739	26083501	
LG36	27130058	27130492	•GRIP and coiled-coil domain-containing protein 2-like, •LIM and senescent cell antigen-like-containing domain protein 1
	27130799	27130958	
LG36	27901792	27903150	•Cytochrome c1-like
Curvilinear velocity (VCL)			
LG3	3581081	3581678	•Hematopoietic lineage cell-specific protein, •Protein LLP homolog
LG6.1	17130126	17130505	•Trophoblast glycoprotein-like, •UBX domain-containing protein 11-like, •Centrosomal protein of 85 kDa, WD and tetratricopeptide repeats protein 1
LG23	11292416	11292625	•Uncharacterized protein LOC111950195, •Natural cytotoxicity triggering receptor 3 ligand 1, •TIP41-like protein, •Ribosomal oxygenase 2, •Tubulin-specific chaperone cofactor E-like protein
LG28	316243	316808	•Coiled-coil domain-containing protein 85C-B-like
LG33	18101554	18101630	•DNA replication complex GINS protein SLD5-like, •Ubiquitin-conjugating enzyme E2 D4, •Phytanoyl-CoA hydroxylase-interacting protein-like, •Drebrin-like protein, •AT-rich interactive domain-containing protein 5 A
	18101782	18101969	

Significant hits retained after Benjamini-Hochberg adjustment are listed.

## Discussion

Spermatogenesis is a fundamental component of sexual reproduction in metazoan organisms and represents a highly complex biological function requiring the coordination of several molecular pathways and physiological mechanisms (Krausz and Riera-Escamilla 2018). In aquaculture settings, sexual maturation and gamete quality is often not only a function of genetic factors but also a result of nutrition (Taranger et al. 2010), light conditions (Duston et al. 2003; Lundova et al. 2019; Al-Emran et al. 2024), temperature trajectories (Vikingstad et al. 2008) and other environmental stressors that compromise physiological processes. Moreover, complex interactions between male and female reproductive factors synthesize to shape the overall success of fertilization and early development (Beirão et al. 2014). In this study, we investigated possible links between fertility indicators in farmed Arctic charr and the DNA methylome of male gametes. Epigenetic marks have previously been suggested to act as early signatures of domestication in fish (Anastasiadi and Piferrer 2019; Podgorniak et al. 2022), with some of them persisting across generations (Rodriguez Barreto et al. 2019). Such mechanisms could possibly explain the observed fertility decline shortly post introduction to captive environments. Interestingly, recent experimental evidence for Chinook salmon (*Oncorhynchus tshawytscha*) supports reversibility and restoration potential for reproduction success rates after only one generation post-reintroduction to the wild (Dayan et al. 2024). Studies in domesticated animals, including salmonid fish, commonly show negative correlations for production traits, such as between growth rate and fertility (Whalen and Parrish 1999; Akanno et al. 2013; Mobley et al. 2021). These relationships are widely believed to reflect trade-offs based on sharing common resources, such as the expectation that mobilization of energy resources towards reproduction can negatively affect growth rate (Stearns 1992; Bernardo 1993; Mobley et al. 2021). Evidence from relevant studies (Gavery et al. 2018; Woods Iii et al. 2018; Brionne et al. 2023; Cheng et al. 2023; Zhang et al. 2023) underscores the need to consider both genetic and epigenetic factors in understanding and managing fertility in aquaculture, highlighting the complex interplay between domestication, environmental adaptation, and reproductive success.

### Methylation landscape and coupling of epigenomic and genomic variation

The Arctic charr sperm genome appears to be hypermethylated, as demonstrated by global methylation levels of around 86%. A similar finding was reported recently in rainbow trout, where the average sperm methylation was 87% (Brionne et al. 2023). Our finding is also in agreement with the consensus on CpG hypermethylation in teleost sperm DNA. Still, more specifically, variation across species might exist since the corresponding proportion of zebrafish sperm is 95% (Potok et al. 2013), 79% in common carp sperm (Cheng et al. 2023, 2024), and 78% in ricefield eel (Chen et al. 2021).

We observed that the highest CpG methylation levels were detected in CpG islands and their shores, most likely serving transcriptomic suppression and genomic stability maintenance. Promoters and first introns, on the other hand, exhibited the most variable methylation levels, indicating their role as genomic switches that guide developmental phenomena through regulating gene expression (Anastasiadi et al. 2018). Although spermatozoa are typically transcriptionally inactive, the methylation profile of sperm DNA is thought to be retained and already established in spermatogonia prior to meiotic divisions, according to investigations in zebrafish (Skvortsova et al. 2019). This profile reflects the transcriptional activity during gamete assembly, supporting the fact that the information for sperm differentiation could be primarily contained within the respective methylome (Ortega-Recalde et al. 2019).

Our results support the hypothesis that methylation is set before the first meiotic division, as indicated by the trimodal CpG methylation pattern in the studied haploid cell populations. This pattern suggests a semiconservative mechanism for the most part, where the corresponding diploid spermatogonia display unmethylated, semimethylated, and hypermethylated states. Additionally, CpG methylation appears regionally linked, showing a non-random association of epialleles and an exponential decay in comethylation with increasing physical distance, similar to linkage disequilibrium of genetic variants. In terms of absolute correlation values, our analysis yielded expected estimates for randomly selected CpGs within intervals of 100 bp (Zhang et al. 2015; Song et al. 2017) that are a bit lower than genomic-context specific correlations (e.g. promoter or CpG island focused studies) and reflect a non-stochastic system that is dependent on the CpG topology across the genome (Lövkvist et al. 2016; Affinito et al. 2020).

Further support for the semiconservative mechanism comes from the comparison of methylation and genetic relationships, where methylation similarities across individuals successfully recovered pedigree structures (Fig. 2). This suggests that in the absence of considerable environmental variation capable of inducing de novo alterations, methylation variation could be attributed to vertical transmission. However, there is still no general consensus on whether epialleles in animals are determined solely by genomic background, inherited and accumulated alongside alleles due to shared environmental factors, or influenced by a combination of both mechanisms (Berbel-Filho et al. 2019; Kelley et al. 2021; Sepers et al. 2023). At the same time, our observations contrast experimental evidence from zebrafish, suggesting that the epigenomic profile in the offspring is, for the most part, set according to the paternal (spermatozoa) and not maternal (oocytes) template (Jiang et al. 2013). As evident in our epigenomic-genomic data (Fig. 2), CpG methylation marks seem to correlate with Mendelian transmission of alleles since paternal half-sibs have lower methylation relationships than full-sibs.

### Associations between DNA methylation and sperm quality traits

Our annotation-dependent and annotation-free approaches to associate CpG methylation marks with sperm quality parameters yielded results tracing the possible underlying biological mechanisms and networks governing male fertility. Comethylation analysis for promoters, CpG islands and first introns successfully reduced the dimensionality of our data and elucidated gene groups that are either upregulated or downregulated in low fertility indicators (Fig. 3). A consistent observation across all genomic features (promoters, CpG islands, and first introns) was the presence of modules showing opposing correlations with sperm concentration and motility/velocity. Despite the relatively low sample size, this pattern suggests a potential trade-off between sperm density and motility, that is mediated by epigenetic regulation and which could be biologically explained by the resource competition between the two traits. We hypothesize that in denser milt, the increased number of spermatozoa may lead to competition for limited resources such as energy and nutrients in the seminal plasma, potentially compromising their motility. Though, some species present a notable seasonal variation in overall sperm quality (Rakitin et al. 1999; Mylonas et al. 2003), usually with a synchronized incline and decline of sperm density and motility (Rouxel et al. 2008). Furthermore, differences in seminal plasma composition, reproductive strategies, or the timing of sperm release could be species-specific. Therefore we recognize that more powerful and targeted study designs are necessary to confirm whether male Arctic charr indeed follow a divergent reproductive strategy.

A notable proportion of the detected genes and gene-enrichment terms had strong biological relevance to reproduction, implying that despite the expected noise from the comethylation networks construction, our analysis probably also highlighted several causative differential methylation loci. Such findings, whose coexpression pointed to various components of spermatogenesis were identified and analyzed under the context of gene-set enrichment analysis. Remarkably, several of the top ranking terms for cellular components, biological processes and molecular pathways concerned cytoskeletal regulation and flagella assembly, cell cycle, endocrine signaling in reproduction and cell motility (Fig. 4). For instance, genes upregulated in low sperm concentration encode proteins that were predominantly associated with heart function, cytoskeleton remodeling, and signaling pathways such as those involved in insulin signaling and inflammation. This suggests a complex interplay between divergent molecular mechanisms involved in sperm production.

On the other hand, genes downregulated in cases of low sperm motility and velocity were found to encode proteins linked to mitochondrial function, energy production and axonemal assembly, suggesting a link between these biological processes and spermatozoa’s ability to move rapidly and effectively. Notably, our study also identified anandamide metabolism, a pathway tightly linked to human sperm motility (Amoako et al. 2013). Furthermore, the upregulated genes in low sperm motility/velocity were enriched in pathways related to spermatogenesis, neural signaling, and cell survival. This could indicate that disruptions in relevant molecular mechanisms could adversely affect spermatozoa.

Lastly, our annotation-free association approach identified some genomic signals associated with sperm concentration and curvilinear velocity. The identified peaks for sperm concentration highlighted specific chromosomal regions that may harbor essential genes influencing this trait. The proximity of the strongest signal to a gene coding for cytochrome c1-like protein, which is involved in cellular respiration and apoptosis (Abramczyk et al. 2022), hints at a potential link between mitochondrial function and sperm concentration. This is particularly interesting given that mitochondria are crucial for energy production, which is vital for spermatogenesis (Medar et al. 2021; Silva et al. 2022). The sequence similarity to *DHRSX* and the presence of other PAR-related genes like *AKAP17A* and *ARSH* in the same region hold promise for further investigation in a comparative manner. In the case of curvilinear sperm velocity (VCL), the epigenetic signals partly overlapped with the ones identified by comethylation analyses and were related to genes involved in DNA replication, ubiquitination, and actin cytoskeleton dynamics. The actin cytoskeleton, in particular, is crucial for maintaining the structural integrity of sperm enabling its movement (Soda et al. 2020) and therefore is directly linked to successful fertilization. Our finding that some signals are co-localized in specific chromosomal segments suggests that these regions may be hotspots for epigenetic regulation of sperm quality. The potential clustering of CpG sites associated with both sperm concentration and velocity raises the possibility that these loci could be critical nodes in the regulatory network governing spermatogenesis and sperm function. This clustering could also imply that multiple genes within these regions might be co-regulated or that there are shared epigenetic mechanisms influencing different aspects of sperm quality. All aforementioned associations underscore the importance of considering the complexity and interconnectedness of various cellular phenomena when studying male reproductive capacity.

## Concluding remarks

DNA methylation profiling of haploid genetic material derived from male gametes provided insights regarding its variation, dynamics and links to genetic and phenotypic variation. Overall, our results indicate that male fertility components in Arctic charr are epigenetically regulated. High-resolution information for cytosine methylation was associated with sperm concentration and kinematic parameters both when considering comethylation networks and groups of co-localized CpGs. Interestingly, further analysis and enrichment of associated epigenetic marks pointed towards biological mechanisms and pathways involved in male fertility. However, the critical question remains whether the sperm epigenome mediates the effects of DNA sequence variation or is environmentally induced to a certain degree and transmitted to offspring. Our study highlights the importance of considering epigenetic factors in male fertility and suggests that profiling of gamete epigenomes might be of value in the future for aquaculture breeding management. Beyond fish farming, the implications of these findings could extend to evolutionary biology by shedding light on mechanisms that drive variation in male reproductive success and possible trade-offs in resource allocation. Lastly, there are also potential applications in conservation efforts, particularly in managing fertility challenges that captive populations face.

## Supplementary information

Supplemental Material

## Data Availability

The sequenced reads were deposited in the National Centre for Biotechnology Information repository as fastq files under project ID PRJNA803600.
