# Supplementary material for: Sperm DNA methylation landscape and its links to male fertility in a non-model teleost using EM-seq
Source: Heredity (Edinb). 2025 Mar 18;134(5):293–305. doi: 10.1038/s41437-025-00756-y (PMC12056074; doi:10.1038/s41437-025-00756-y)
Supplement: Supplementary file 1 — Supplemental Material [file 41437_2025_756_MOESM1_ESM.docx]

**Supplementary material**

**Table S1.** Methylation levels of sequenced Arctic charr sperm libraries

| **Sample Nr** | **Sample Id** | **Acccesion numbers** | **CpG** | **CHH** | **CHG** | **Unknown context** | **CpG in unmethylated control** | **CpG in methylated control** |
| --- | --- | --- | --- | --- | --- | --- | --- | --- |
| 1 | AC-s-2020-293 | SAMN25660337 | 86,4% | 0,3% | 0,3% | 4,9% | 0,1% | 97,5% |
| 2 | AC-s-2020-294 | SAMN25660338 | 85,2% | 0,3% | 0,3% | 4,8% | 0,1% | 97,8% |
| 3 | AC-s-2020-300 | SAMN25660339 | 85,6% | 0,3% | 0,3% | 4,7% | 0,1% | 97,3% |
| 4 | AC-s-2020-304 | SAMN25660340 | 84,8% | 0,3% | 0,3% | 5,0% | 0,1% | 96,9% |
| 5 | AC-s-2020-309 | SAMN25660349 | 86,6% | 0,3% | 0,3% | 5,3% | 0,1% | 97,6% |
| 6 | AC-s-2020-313 | SAMN25660350 | 86,0% | 0,3% | 0,3% | 4,9% | 0,1% | 96,8% |
| 7 | AC-s-2020-320 | SAMN25660341 | 85,1% | 0,3% | 0,3% | 4,7% | 0,1% | 97,7% |
| 8 | AC-s-2020-322 | SAMN25660351 | 85,8% | 0,3% | 0,3% | 4,8% | 0,1% | 97,7% |
| 9 | AC-s-2020-326 | SAMN25660342 | 86,0% | 0,3% | 0,3% | 4,9% | 0,1% | 97,9% |
| 10 | AC-s-2020-336 | SAMN25660352 | 86,2% | 0,3% | 0,3% | 4,8% | 0,1% | 97,8% |
| 11 | AC-s-2020-337 | SAMN25660343 | 86,5% | 0,4% | 0,4% | 4,9% | 0,2% | 97,8% |
| 12 | AC-s-2020-338 | SAMN25660344 | 86,6% | 0,3% | 0,4% | 5,1% | 0,1% | 97,4% |
| 13 | AC-s-2020-339 | SAMN25660353 | 86,3% | 0,3% | 0,3% | 4,9% | 0,1% | 97,7% |
| 14 | AC-s-2020-346 | SAMN25660345 | 85,8% | 0,3% | 0,3% | 5,0% | 0,1% | 97,3% |
| 15 | AC-s-2020-347 | SAMN25660346 | 85,6% | 0,3% | 0,3% | 4,8% | 0,1% | 97,8% |
| 16 | AC-s-2020-348 | SAMN25660354 | 86,2% | 0,3% | 0,3% | 5,0% | 0,1% | 97,8% |
| 17 | AC-s-2020-351 | SAMN25660355 | 86,0% | 0,3% | 0,3% | 4,7% | 0,1% | 98,0% |
| 18 | AC-s-2020-352 | SAMN25660347 | 85,0% | 0,3% | 0,3% | 5,0% | 0,1% | 97,6% |
| 19 | AC-s-2020-353 | SAMN25660356 | 86,2% | 0,3% | 0,3% | 5,2% | 0,1% | 97,4% |
| 20 | AC-s-2020-368 | SAMN25660348 | 85,9% | 0,3% | 0,3% | 5,0% | 0,1% | 97,7% |
| 21 | AC-s-2020-378 | SAMN25660357 | 85,9% | 0,3% | 0,3% | 4,8% | 0,1% | 97,8% |
| 22 | AC-s-2020-379 | SAMN25660358 | 86,1% | 0,3% | 0,3% | 4,9% | 0,1% | 97,5% |
| 23 | AC-s-2020-412 | SAMN25660359 | 85,9% | 0,3% | 0,3% | 4,8% | 0,1% | 97,4% |
| 24 | AC-s-2020-414 | SAMN25660360 | 86,6% | 0,3% | 0,4% | 4,8% | 0,2% | 98,1% |
| 25 | AC-2017-s-373 | SAMN25660377 | 85,7% | 0,3% | 0,3% | 4,4% | 0,1% | 97,3% |
| 26 | AC-2017-s-374 | SAMN25660378 | 85,3% | 0,3% | 0,3% | 4,8% | 0,1% | 97,4% |
| 27 | AC-2017-s-380 | SAMN25660379 | 84,9% | 0,3% | 0,3% | 4,6% | 0,1% | 97,7% |
| 28 | AC-2017-s-384 | SAMN25660380 | 84,2% | 0,3% | 0,3% | 4,7% | 0,1% | 97,0% |
| 29 | AC-2017-s-386 | SAMN25660381 | 85,3% | 0,3% | 0,3% | 4,7% | 0,1% | 97,4% |
| 30 | AC-2017-s-387 | SAMN25660382 | 85,4% | 0,3% | 0,3% | 4,5% | 0,1% | 97,4% |
| 31 | AC-2017-s-388 | SAMN25660361 | 84,5% | 0,3% | 0,3% | 4,6% | 0,1% | 97,3% |
| 32 | AC-2017-s-389 | SAMN25660362 | 85,5% | 0,3% | 0,3% | 4,8% | 0,1% | 97,8% |
| 33 | AC-2017-s-390 | SAMN25660363 | 84,1% | 0,3% | 0,3% | 4,7% | 0,1% | 97,3% |
| 34 | AC-2017-s-391 | SAMN25660364 | 86,3% | 0,3% | 0,3% | 4,6% | 0,1% | 97,9% |
| 35 | AC-2017-s-394 | SAMN25660365 | 86,0% | 0,3% | 0,3% | 4,6% | 0,1% | 97,8% |
| 36 | AC-2017-s-395 | SAMN25660366 | 85,8% | 0,3% | 0,3% | 4,5% | 0,1% | 97,8% |
| 37 | AC-2017-s-396 | SAMN25660367 | 85,4% | 0,3% | 0,3% | 4,8% | 0,1% | 97,6% |
| 38 | AC-2017-s-397 | SAMN25660368 | 85,4% | 0,3% | 0,3% | 4,6% | 0,1% | 97,7% |
|  | **AC-2017-s-398*** | SAMN25660369 | **97,2%** | **89,2%** | **89,5%** | **75,4%** | **88,3%** | **99,8%** |
| 39 | AC-2017-s-399 | SAMN25660370 | 85,8% | 0,3% | 0,3% | 4,5% | 0,1% | 97,1% |
| 40 | AC-2017-s-402 | SAMN25660371 | 85,5% | 0,3% | 0,3% | 4,7% | 0,1% | 96,9% |
| 41 | AC-2017-s-403 | SAMN25660372 | 86,0% | 0,3% | 0,3% | 4,7% | 0,1% | 97,1% |
| 42 | AC-2017-s-405 | SAMN25660373 | 84,1% | 0,3% | 0,3% | 4,4% | 0,1% | 97,8% |
| 43 | AC-2017-s-408 | SAMN25660374 | 85,1% | 0,3% | 0,3% | 5,0% | 0,1% | 96,9% |
| 44 | AC-2017-s-410 | SAMN25660375 | 85,1% | 0,3% | 0,3% | 4,9% | 0,1% | 98,1% |
| 45 | AC-2017-s-415 | SAMN25660376 | 85,4% | 0,3% | 0,3% | 4,8% | 0,1% | 97,5% |
| 46 | AC-2017-s-416 | SAMN25660383 | 85,7% | 0,3% | 0,3% | 4,4% | 0,1% | 98,5% |
| 47 | AC-2017-s-417 | SAMN25660384 | 85,9% | 0,3% | 0,3% | 4,6% | 0,0% | 97,8% |

| **^*^** ^this sample was removed from downstream analysis^ |
| --- |


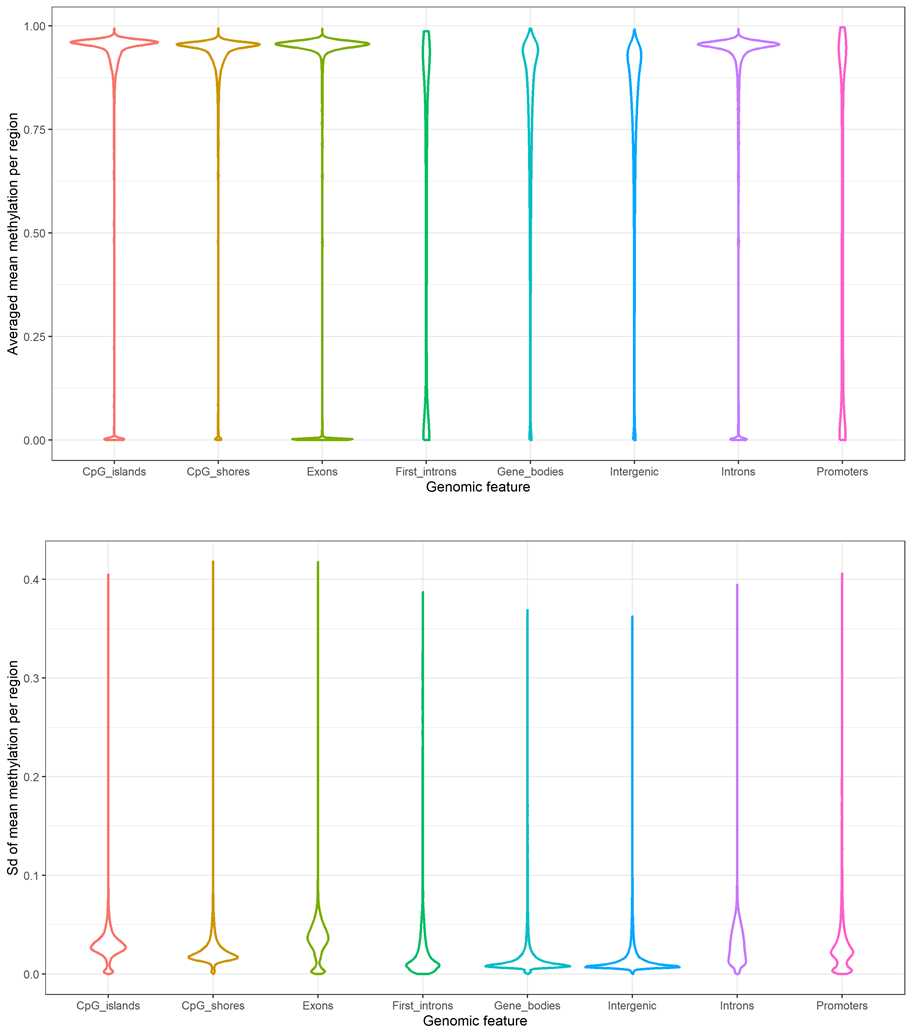


**Figure S1:** Violin plots visualising distributions of averaged mean methylation per region and standard deviation of mean methylation per region. Separate violins are plotted for the following genomic feature sets: CpG islands, CpG island shores, exons, first introns, gene bodies, intergenic regions, introns and promoters defined as 1Kbp regions upstream of gene bodies. The figure was created with R/ggplot2 v3.4.4 (Wickham, 2016).


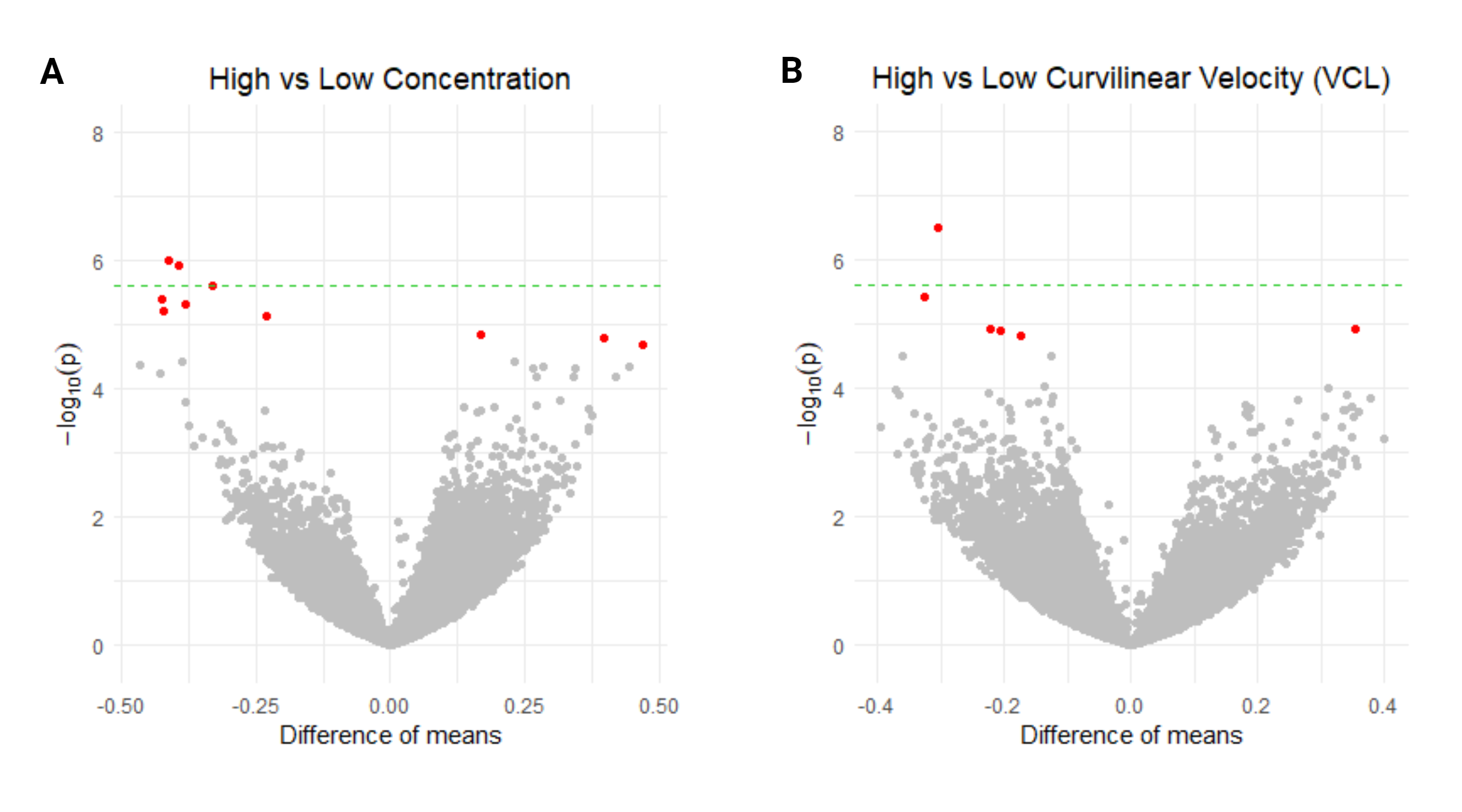


**Figure S2:** Volcano plots of methylome association scans for **A)** sperm concentration and **B)** curvilinear velocity (VCL)**.** Differences between the methylation means for CpGs in respective high and low groups are considered along the horizontal axis while the vertical axis corresponds to the negative decimal logarithms for the p-values yielded by the t-tests. Dashed green line defines the $-{log}_{10}a$ of the Bonferroni adjusted significance threshold (5%). Associations found significant after the Benjamini-Hochberg correction are marked with red colour. The figure was created with R/ggplot2 v3.4.4 (Wickham, 2016).
